# Comparison of traditional template measurements and artificial intelligence preoperative planning in total knee arthroplasty

**DOI:** 10.3389/fsurg.2025.1573148

**Published:** 2025-04-15

**Authors:** Meipeng Min, Xiaotian Wang, Rafi Urba, Wenjie Zhang, Jia Gao, Lei Fan

**Affiliations:** Department of Orthopedics, The Second Affiliated Hospital of Nanjing Medical University, Nanjing, China

**Keywords:** total knee arthroplasty, artificial intelligence, preoperative planning, template measurement, prosthesis

## Abstract

**Background:**

The poor reliability of preoperative planning measured by traditional x-ray templates increases the difficulty of osteotomy and prosthesis implantation during an operation, which to some extent affects the surgical outcome of total knee arthroplasty and postoperative satisfaction of patients.

**Objective:**

To evaluate the accuracy and effectiveness of artificial intelligence (AI) preoperative planning in total knee arthroplasty (TKA).

**Methods:**

We prospectively selected 48 patients who underwent primary total knee arthroplasty for knee osteoarthritis in our Joint Surgery Department between March 2021 and May 2022. The test group included 24 patients who underwent three-dimensional preoperative planning using artificial intelligence (AI), and the control group consisted of 24 patients who underwent two-dimensional preoperative planning using traditional template measurement. The differences were not statistically significant when comparing the general information of the two groups, such as gender, age, BMI, affected side category, ASA classification, history of diabetes, history of stroke (*P* > 0.05). For analyzing the accuracy and application effect of the two preoperative planning methods, the intraoperative operation time, intraoperative blood loss, postoperative drainage volume, postoperative lower limb alignment angle, VAS score, and AKS score were compared between the two groups.

**Results:**

All patients were followed for 6–8 months, and no postoperative complications or postoperative deaths occurred in either group. There was no statistically significant difference between the general data of patients in both groups (*P* > 0.05). The complete matching rates of femoral component, tibial component, and tibial liner in the test group were significantly better than those in the control group (*P* < 0.05). The operation time, intraoperative blood loss, and postoperative drainage volume in the test group were significantly less than those in the control group (*P* < 0.05). There was a statistically significant difference in the postoperative lower limb alignment Angle between the two groups (*P* < 0.05). The VAS score of the test group was significantly better than that of the control group within 2 weeks (*P* < 0.05), but there was no statistically significant difference after 1 month (*P* > 0.05). The AKS score of the test group was significantly higher than that of the control group at 3 months after operation (*P* < 0.05).

**Conclusion:**

Compared with traditional film planning, AI preoperative planning can improve the accuracy of intraoperative prosthesis implantation and the surgical outcome of TKA, which is worthy of further promotion and application in clinical practice.

## Introduction

Since the development of total knee arthroplasty (TKA) in the 1960s, and then widely used in clinical practice in the 1970s and 1980s, it has significantly improved patient's postoperative mobility and quality of life by restoring the alignment and soft tissue balance of the knee. It has been demonstrated to be a successful and effective approach for treating knee pain and deformity and is also one of the most successful orthopedic surgeries (1).

The traditional preoperative planning of TKA is carried out under two-dimensional conditions, and the orthopedic surgeon evaluates the patient's lower limb alignment and deformity based on preoperative x-ray images and simply measures the osteotomy during the operation. This method of preoperative planning is rough, inaccurate, and unreliable, providing limited help to clinicians. The intraoperative positioning, osteotomy angle, osteotomy amount, prosthesis size, and soft tissue balance depend on the surgeon's clinical experience and the osteotomy positioning device used (2). Poor force line alignment, poor rotation alignment, and mismatched prosthesis size during surgery can alter the biomechanical properties of the knee joint (3) and result in postoperative knee instability, poor patellar trajectory, loose sterility of the prosthesis and increased wear of the polyethylene liner (4), which greatly affect the accuracy and effectiveness of the operation. More than half of the early failures of TKA are related to improper placement of the prosthesis and improper alignment (5, 6), resulting in patient satisfaction in only 80% after the operation (7). In addition, multiple intramedullary positions during TKA prolong the operation time and increase bleeding (8), which can increase the risk of intraoperative infection and affect the postoperative recovery of patients (9).

With the advancements in artificial intelligence (AI) and its gradual application in TKA, CT, MRI, and other imaging data are now available for reconstructing a three-dimensional model of the knee joint before surgery, and a visual osteotomy scheme is provided, allowing the surgeon to observe the anatomical points of the knee joint clearly (10). In addition, according to the characteristics and lesions of the patient's knee joint, surgeons can select the appropriate osteotomy method, prosthesis size, implant position and angle when simulating the surgical operation of the knee joint model to improve the stability of the prosthesis after implantation, restore the anatomical structure and kinematic characteristics of the affected knee joint under a normal physiological state to the greatest extent possible (11), and avoid poor surgical results caused by poor prosthesis matching or implantation deviation. It is helpful to shorten the operation time, reduce intraoperative trauma, and greatly improve the accuracy and safety of osteotomy and the surgical outcome of TKA (12).

The purpose of this study was to compare and analyze the effectiveness of traditional template preoperative planning and artificial intelligence (AI) preoperative planning in TKA, as well as to investigate the accuracy and application potential of AI preoperative planning as a preoperative prosthesis selection plan, to provide a theoretical basis for this process.

## Materials and methods

The study was approved by the Ethics Committee of the Second Affiliated Hospital of Nanjing Medical University. All patients provided informed consent for the treatment regimen and signed informed consent for treatment.

This was a prospective study. The study enrolled 48 patients who underwent primary total knee replacement on the affected side in the same treatment group of the Department of Joint Surgery of the Second Affiliated Hospital of Nanjing Medical University between March 2021 and May 2022. The general data of the patients, such as sex, age, BMI, affected side category, American Society of Anesthesiologists (ASA) classification, history of diabetes, and history of stroke were recorded. The patients were randomly divided into a test group (*n* = 24) using AI three-dimensional preoperative planning and a control group (*n* = 24) using traditional template two-dimensional preoperative planning. Random grouping method: Patients were allocated using a computer-generated randomization sequence (block randomization, 1:1 ratio) stratified by age and BMI. Surgeons were aware of the planning method, but outcome assessors were blinded to group allocation during follow-up. All operations were performed by the same surgeon.

The inclusion criteria for patients were as follows: (1) had a clinical diagnosis of primary osteoarthritis; (2) were older than 50 years and underwent primary TKA; (3) had no cerebrovascular or neurological disease; (4) could tolerate anesthesia after preoperative evaluation by anesthesiologists and had physical conditions that could be tolerated surgery.

The exclusion criteria for patients were as follows: (1) were under 50 years old; (2) had autoimmune diseases such as rheumatoid arthritis; (3) had severe underlying disease and could not tolerate surgery; (4) had central nervous system disease (affecting postoperative knee function score); (5) underwent revision knee arthroplasty; (6) had incomplete medical records and failed to follow up regularly; (7) were unable to follow the doctor's advice for functional rehabilitation and postoperative recovery.

Imaging examinations Patients in both groups underwent full-length x-rays of both lower limbs before the operation, 24 patients in the control group underwent frontal and lateral x-rays of the knee joint, and 24 patients in the test group underwent a CT scan of the knee joint.

The CT scan parameters were as follows: voltage, 120 kV; layer thickness, 1 mm; and matrix, 512 × 512. The patient was lying flat on the examination table, both lower limbs were kept in a straight and neutral position, and both patellae were suprapatellar. The scanning range was from the center of the femoral head to the center of the ankle joint, and the scanned data were stored and exported in DICOM format.

Two-dimensional planning of traditional template measurements The film template provided by the prosthesis manufacturer was used to measure and compare frontal and lateral x-ray images of the knee joint to predict the size and insertion angle of the prosthesis during the operation.

### AI computer-aided 3D planning

(1) Data import: The CT data of the KNEE joint were imported into the AI 3D total knee joint planning system software (AI KNEE, Beijing Changmugu Medical Technology Co., Ltd.) in DICOM format.
(2) Intelligent segmentation: artificial intelligence (AI) technology was used to automatically segment the bones; create 3D models of the entire length of the lower limb, femur, and tibia; and measure the mechanical axis of the lower limb, femoral anatomical axis, tibial anatomical axis, femoral external rotation angle, tibial posterior slope angle, and other parameters.
(3) Femoral component planning: ① The femoral opening point, femoral valgus angle, and femoral external rotation angle were measured; ② The osteotomy thickness was assessed to determine the size, position, and angle of the femoral component.
(4) Tibial component planning: ① The tibial plateau osteotomy thickness and posterior slope angle were measured; ② The size, rotation, and coverage of the tibial prosthesis were all planned.
(5) Completion of planning: The femoral component, tibial component, and tibial liner were all shown in place, and the postoperative effects were simulated.

**Figure 1:**
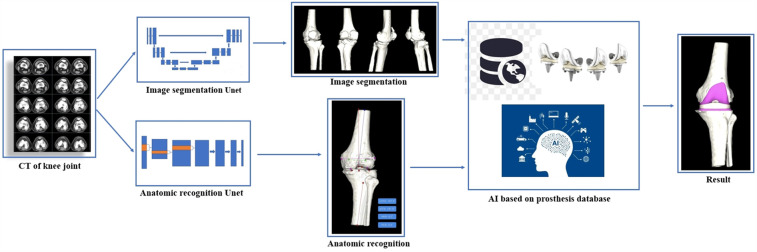
Flowchart of how the AI KNEE works.

**Figure 2:**
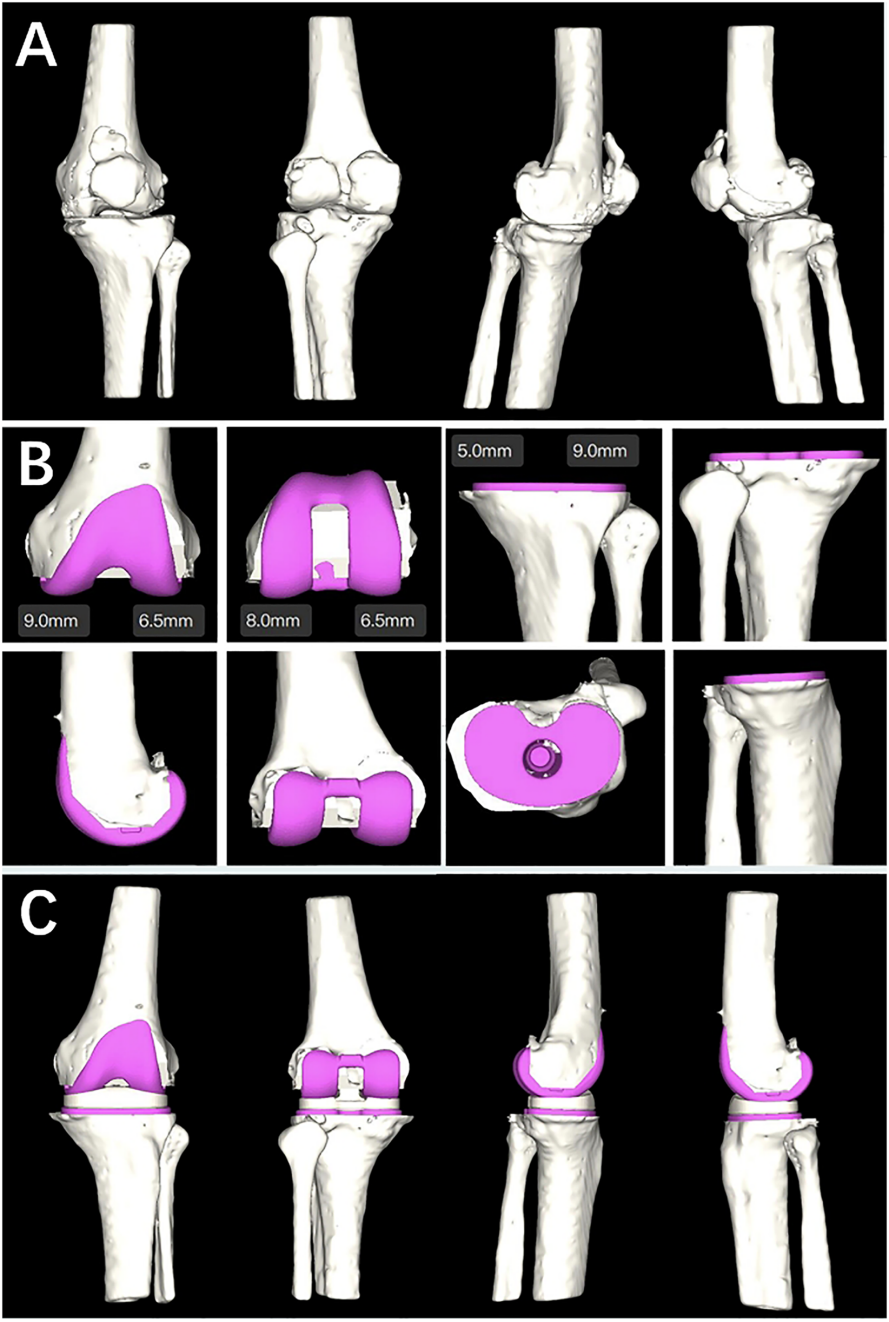
**(A)** Ai KNEE modeling; **(B)** completion planning of the femoral component, tibial component and tibial liner; **(C)** simulation of postoperative effects.

The working principle of the AI KNEE is shown in Figures 1, 2.

**Figure 3:**
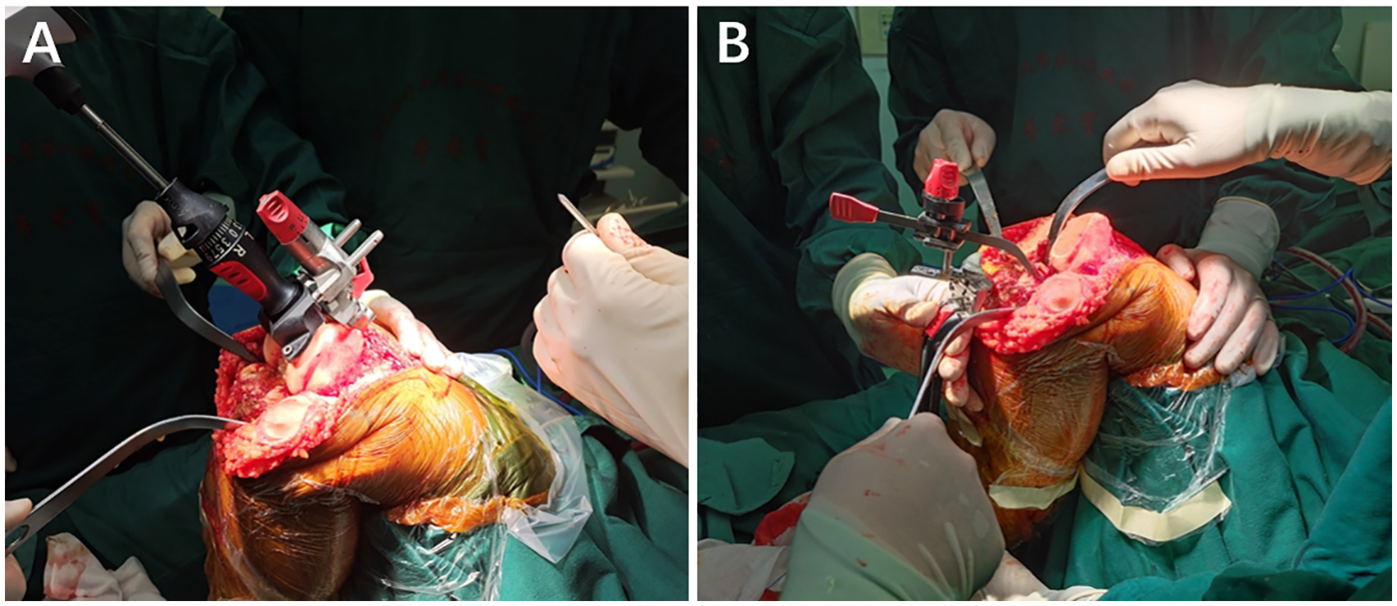
**(A)** Measurement of the distal femur; **(B)** measurement of the proximal tibia.

Surgical methods All surgeries were performed by the same senior professional title surgeon in the Department of Joint Surgery, the Second Affiliated Hospital of Nanjing Medical University. The surgeon was aware of the implant size predicted by the AI KNEE and x-ray film templates. Following general or epidural anesthesia, the patient was supine, and an inflatable tourniquet was inserted at the root of the thigh of the afflicted knee. The injured leg was routinely sterilized with povidone-iodine, and a sterile towel sheet was used. The tourniquet was inflated to 40 Kpa, and a 10-cm longitudinal incision was made in the middle of the anterior knee joint. The subcutaneous tissues were incised, and the joint capsule was incised using the medial parapatellar approach to reveal the knee joint. The synovial tissue and several fat pads were removed, and the anterior and posterior cruciate ligaments were severed. Cuff dissection revealed the articular surface of the tibial plateau, loosened the medial collateral ligament stop, removed the joint cavity, and resected the medial and lateral menisci, anterior and posterior cruciate ligaments, and hyperplastic tissues. The femoral osteotomy was performed intramedullary, and the medulla was opened 0.8–1 cm above the posterior cruciate ligament's stopping point. The distal end of the femur was severed 9 mm by 6° external rotation. The tibia was positioned extramedullary, and the bone was severed 9 mm along the tibial plateau at a 3° posterior angle. The osteotomy gap was estimated accurately. The osteotomy gap was estimated accurately. The size of the femoral condyles was determined, and a 4-in-1 osteotomy plate was used to perform a 3° externally rotated osteotomy and an intercondylar osteotomy. The medial and lateral meniscus, the posterior cruciate ligament, and the hyperplastic tissue were all removed. The curved osteotome removes the osteochondral tuberosity of the posterior femoral condyle, loosens the posterior joint capsule, and installs a trial mold to test the joint's stability and mobility by using the gap balancing method before drilling holes to shape the tibial plateau once the results are satisfactory. The patella was trimmed, and the choice to replace the patellar prosthesis was made based on the amount of patellar cartilage wear and thickness. The joint cavity was flushed with saline, the bone cement was blended, and the tibial (ATTUNE-RP) and femoral (ATTUNE-PS) prostheses were installed, with a polyethylene liner (ATTUNE-PS-RP) applied after the bone cement was fixed. After the bone cement had firm, the joint cavity was cleaned with saline and dilute iodophor, a negative pressure drain was implanted, and the surrounding soft tissues were injected with a ropivacaine and betamethasone mixture before being sutured layer by layer. The intraoperative picture is shown in Figure 3.

Postoperative management After the operation, the negative pressure drainage tube and autologous blood transfusion system were placed in the knee joint of the two groups, which was removed after 48 hours, and anti-infection treatment was administered for 24 hours. On the second day following the procedure, quadriceps femoris functional exercise and knee flexion and extension exercises were performed on the second day after the operation, and bed standing exercise was performed on the third day. Anteroposterior and lateral radiographs of the knee joint were taken routinely to evaluate the relationship between the prosthesis and the alignment.

### Data collection

(1) Patient-related data included sex, age, BMI, affected side category, ASA classification, history of diabetes, and history of stroke.
(2) Surgery-related data ① The actual prosthesis used during the operation was matched with the preoperative planning prosthesis size in the two groups. The preoperative plan was defined as accurate when the preoperative planning model was completely consistent with the actual application model during the operation. ② Intraoperative operation time (unit: min), intraoperative blood loss (unit: ml), and postoperative drainage volume (unit: ml) in the two groups. ③ Absolute deviation values of the postoperative lower limb alignment angle and standard angle of patients in the two groups (unit:°): The coronal hip-knee angle (HKA), frontal femoral component (FFC), and frontal tibial component (tibial component) angles were measured. The FTC, lateral femoral component (LFC), and lateral tibial component (LTC) angles on the sagittal plane and the absolute deviation from the standard angle were recorded. The absolute deviation between the actual postoperative lower limb alignment and a standard angle ≤3° was considered good.
(3) Postoperative follow-up data: ① Pain visual analog scale (VAS) scores before the operation and at 1 day, 1 week, 2 weeks, 1 month, and 3 months after the operation in the two groups. ② Knee joint function score (AKS score) of the two groups 3 months after the operation.

### Statistical analysis

SPSS 26.0 statistical software was used to process and analyze the data. Continuous variables were reported using averages and standard deviations (SD) and categorical variables were presented using frequency distributions and percentages. For categorical variables, the chi-squared test was employed to assess differences in proportions between the two groups. For continuous variables, the Mann–Whitney *U*-test was used to compare the medians between groups. *P* < 0.05 was considered to indicate statistical significance. Biostatisticians from The Second Affiliated Hospital of Nanjing Medical University reviewed the statistical methods used.

## Results

### Number of subjects analysis

All 48 patients underwent successful surgery, and no intraoperative or postoperative complications occurred in either the control group or test group. All patients were followed up for 6 to 8 months, with an average of 7.5 months. The prosthesis implanted in this study had good biocompatibility, and no adverse reactions from the material host occurred in any of the patients.

A comparison of the baseline data of the patients is shown in Table 1.

**Table 1:** Comparison of baseline data between the two groups (*n* = 24).

Variables	Test group (*N* = 24)	Control group (*N* = 24)	*P* value
Sex (*n*)			0.771
Male	10	11	
Female	14	13	
Age (x̅ ± s, years)	74.6 ± 4.2	72.3 ± 4.8	0.084
BMI (x̅ ± s, kg/m^2^)	24.7 ± 3.6	25.2 ± 3.4	0.623
Affected side category (*n*)			0.773
Left	11	12	
Right	13	12	
ASA grade (*n*)			0.947
Ⅰ	5	4	
Ⅱ	10	11	
Ⅲ	7	8	
Ⅳ	2	1	
History of diabetes (*n*)			0.386
Yes	10	13	
No	14	11	
History of stroke (*n*)			0.383
Yes	12	9	
No	12	15	

There was no statistically significant difference in the general data between the two groups (*P* > 0.05).

### Comparison of the accuracy of AI 3d planning and traditional template measurements

The accuracy of AI 3D planning of the femoral component was 91.67% (22/24), and the accuracy of film template 2D planning was 66.67% (16/24). The difference between the two groups was statistically significant (*P* < 0.05). The accuracy of 3D planning of the tibial component AI was 87.50% (21/24), the accuracy of 2D preoperative planning of the film template was 62.50% (15/24), and the difference was statistically significant (*P* < 0.05). The accuracy rate of 3D planning for tibial liner AI was 91.67% (22/24), and the accuracy rate for 2D planning of film templates was 62.50% (15/24). The difference between the two groups was significant (*P* < 0.05). The specific data are shown in Table 2.

**Table 2:** Comparison of the two types of prostheses used during surgery and preoperative planning [*n*(%)*, n* = 24].

Type of prosthesis	Test Group (*N* = 24)		Control Group (*N* = 24)		X ²	*P* value
	Exactly in line with (n)	Coincidence rate (%)	Exactly in line with (n)	Coincidence rate (%)		
Femoral component	22	91.67%	16	66.67%	4.547	0.033
Tibial component	21	87.50%	15	62.50%	4.000	0.046
Tibial liner	22	91.67%	15	62.50%	5.779	0.016

### Comparison of intraoperative operation time, intraoperative blood loss, and postoperative drainage

Intraoperative operation time and intraoperative blood loss between the two groups. The AI three-dimensional preoperative planning operation time was 68.2 ± 10.6 (min), and the operation time of film template two-dimensional preoperative planning was 84.5 ± 11.4 (min). The difference was statistically significant (*P* < 0.05). The intraoperative blood loss during three-dimensional AI preoperative planning was 110.5 ± 20.3 ml, that two-dimensional film template preoperative planning was 164.5 ± 43.7 ml, and the difference was statistically significant (*P* < 0.05). The postoperative drainage volume of the AI 3D preoperative plan was 182.4 ± 23.8 ml, and that of the film template 2D preoperative plan was 266.8 ± 37.1 ml. There was a significant difference between the two groups (*P* < 0.05). The test group was superior to the control group. The specific data are shown in Table 3.

**Table 3:** Comparison of intraoperative operation time, intraoperative blood loss, and postoperative drainage volume between the two groups (*x̅ ± s, n* = 24).

Items	Test group	Control group	T value	*P* value
Operation time (min)	68.2 ± 10.6	84.5 ± 11.4	−5.130	<0.001
Intraoperative blood loss (ml)	110.5 ± 20.3	164.5 ± 43.7	−5.490	<0.001
Postoperative drainage volume	182.4 ± 23.8	266.8 ± 37.1	−9.381	<0.001

### Postoperative comparison of lower limb force line angles, and absolute deviation from ideal angles

There were statistically significant differences in HKA, FFC, FTC, LFC, and LTC angles between the two groups after the operation (*P* < 0.05). The reconstruction effect of lower limb alignment was significantly better for preoperative planning via AI than for preoperative planning via the film template (*P* < 0.05). The specific data are shown in Table 4.

**Table 4:** Comparison of postoperative lower limb force between the two groups (*x̅ ± s*, *n* = 24) and comparisons of absolute deviation (*n* = 24).

Items	Test group	Control group	T value	*P* value
HKA	181.2° ± 1.4°	178.6° ± 2.5°	4.445	<0.001
FFC	90.3° ± 1.9°	88.5° ± 2.9°	2.544	0.014
FTC	89.4° ± 2.1°	91.8° ± 2.3°	−3.775	0.001
LFC	88.7° ± 1.6°	87.1° ± 2.5°	2.641	0.011
LTC	87.9° ± 1.4°	86.4° ± 1.7°	3.337	0.002
			X²	*P* value
Absolute deviation value ≤3° (*n*)	22	16	4.547	0.033
Absolute deviation >3° (*n*)	2	8		

**Table 5:** Comparison of postoperative VAS scores between the two groups (*x̅ ± s, n* = 24).

Items	Test group	Control group	T value	*P* value
Preoperatively	7.8 ± 2.3	8.0 ± 1.7	−0.343	0.734
1 Day	5.1 ± 1.3	6.0 ± 1.5	−2.221	0.031
1 week	3.8 ± 1.1	4.4 ± 0.8	−2.161	0.036
2 weeks	3.0 ± 1.2	3.8 ± 1.3	−2.215	0.032
1 month	2.3 ± 0.6	2.4 ± 1.1	−0.391	0.670
3 months	1.2 ± 0.3	1.1 ± 0.5	0.840	0.405

### Comparison of visual analog scale (VAS) scores before and after surgery

The visual analog scale (VAS) score for preoperative planning via the AI was significantly better than that for preoperative planning via the film template at 1 day, 1 week, and 2 weeks after the operation in both groups (*P* < 0.05). There was no significant difference in VAS score between the two groups before the operation, or at 1 month, or 3 months after the operation (*P* > 0.05). The specific data are shown in Table 5.

### Comparison of knee function score (AKS) between the two groups of patients three months after surgery

The AKS score of the AI preoperative planning group was significantly greater than that of the film template preoperative planning group three months after the operation (*P* < 0.05). The specific data are shown in Table 6.

**Table 6:** Comparison of the AKS score between the two groups at three months after the operation (*x̅ ± s, n* = 24).

Items	Test group	Control group	T value	*P* value
Joint score	91.74 ± 3.43	86.36 ± 4.84	4.443	<0.001
Pain	44.69 ± 2.75	42.38 ± 2.66	2.958	0.005
Range of motion	23.71 ± 3.52	21.66 ± 3.44	2.041	0.047
Stability	22.37 ± 2.31	20.67 ± 2.89	2.251	0.029
Functional rating	90.84 ± 4.16	85.14 ± 3.73	5.000	<0.001
Walking	44.65 ± 3.85	42.67 ± 2.63	2.080	0.043
Up and down stairs	44.21 ± 4.15	41.37 ± 3.79	2.476	0.017

## Discussion

Total knee arthroplasty (TKA) is one of the most commonly used and effective methods for treating end-stage knee osteoarthritis. According to statistics from the World Health Organization, the prevalence of knee osteoarthritis in the elderly population older than 75 years is more than 80%, and the disability rate of knee osteoarthritis worldwide is as high as 53%. Approximately 94%–97% of all TKA cases are due to osteoarthritis. At present, there are more than 300 million osteoarthritis patients worldwide (13). With the increasing degree of aging in the global population, the prevalence of osteoarthritis is gradually increasing, and the number of TKA surgeries is expected to increase exponentially worldwide. However, many factors are difficult to control during TKA, such as the recovery of lower limb alignment, the selection of prosthesis, the implant position, and the soft tissue balance (14). If the operation is not performed properly, it is easy to cause various postoperative complications, such as aseptic loosening of the prosthesis, infection, pain, patellofemoral joint instability, and poor knee joint activity (15, 16). As a result, approximately 20% of patients were unsatisfied with the efficacy of TKA and the short life of the prosthesis (17). More accurate preoperative planning is needed to assist surgeons in achieving more accurate alignment, perfect soft tissue balance, and superior prosthetic matching, as well as longer prosthesis life, better postoperative function, and greater patient satisfaction.

The preoperative planning of traditional TKA is not only affected by subjective factors such as the surgeon's visual acuity and experience but also by individual differences (18), such as incorrect rotation, flexion, and posture of the affected limb during x-ray imaging or accompanied by knee dysplasia, severe varus and valgus deformity, joint instability and other factors (19), resulting in unclear bone landmark imaging. It is easy to cause measurement errors, which can lead to certain difficulties in accurate positioning, osteotomy, and prosthesis selection and implantation. In addition, the knee joint is surrounded by muscles and soft tissues, and determining its anatomical landmarks is difficult when x-ray images are taken (20). Although the accuracy of osteotomy and prosthesis implantation has improved by the continuous improvement of a mechanical positioning system, the inherent limitations of the mechanical positioning system limit its accuracy (21), which is also the main cause of prosthesis implantation deviation and malalignment. Artificial intelligence (AI) preoperative planning is based on CT scan images of the patient's joint, and uses image processing technology to establish an accurate three-dimensional model of the knee joint and fully expose the anatomical landmarks of the knee joint to compensate for the shortcomings of traditional preoperative planning (22). Artificial intelligence can assist surgeons in determining the anatomical position of osteotomy, the direction, and the angle of prosthesis implantation; measuring the biological force line of the lower limbs; and analyzing the surgical effect under the computer's three-dimensional visualization environment to determine the best surgical plan, improve surgical accuracy, reduce postoperative complications, prolong the life of the prosthesis, and improve postoperative satisfaction (23).

This study compared the clinical efficacy of film template measurements and artificial intelligence (AI) methods applied in TKA preoperative planning. The results demonstrated that, when compared to traditional TKA preoperative planning, AI preoperative planning can not only accurately predict prosthesis size and the implantation direction and angle but also reduce the difficulty of the operation, improve the safety and accuracy of osteotomy, and achieve the best prosthesis alignment. It can also reduce the operation time, intraoperative blood loss, postoperative drainage volume, and incidence of postoperative infections and significantly improve the effectiveness of early postoperative rehabilitation. Obviously, it is based on accurate preoperative planning that reduces the time of osteotomy, model testing, and implant placement, thus shortening the operation time and intraoperative drainage volume, and reducing the trauma of patients from surgery. In addition, the more accurate preoperative planning of the test group in this study made the postoperative reconstruction of lower limb alignment and functional recovery of the knee joint better than that of the control group. Consistent with the results of this study, Lambrechts (24) et al. collected 5,409 TKA patients for preoperative planning. Compared with the actual prosthesis sizes used by orthopedic surgeons during surgery, the complete coincidence rates of femoral and tibial prosthesis sizes planned by instrument manufacturers using film templates were 68.4% and 73.1%, respectively. Artificial intelligence preoperative planning can reduce the time spent on planning and surgery by 39.71%, improve surgical accuracy, and reduce the average correction required by the surgeon. Pietrzak (25) et al. conducted a retrospective study on 31 patients who underwent TKA. Compared with the actual prosthesis size during surgery, the accuracy of the femoral prosthesis 3D template and film template was 96.6% and 52.9%, respectively. The accuracy of the tibial prosthesis 3D template and film template was 93.1% and 28.7%, respectively. It is considered that 3D preoperative planning can reduce the incidence of malalignment of the lower limbs and malalignment of the prosthesis, thereby reducing the risk of infection, and has the potential to prolong the life of the prosthesis. In addition, there is no need for additional learning and training, the purchase of equipment or systems, additional maintenance or consumables, and the ability to greatly reduce medical costs. As a result, AI-based preoperative planning has an outstanding cost-performance ratio compared to traditional template planning.

Robot-assisted surgery is an important area of artificial intelligence applications and is divided into image-based or image-less options. Specifically, the image-based option uses 2D x-rays that are transformed into a digital 3D replication of the patient's anatomy, while the image-less system relies entirely on the acquisition of intraoperative landmarks. Various surgical techniques can be employed, including measured resection, gap balancing, functional alignment, and kinematic alignment (26). In this study, the AI KNEE software for preoperative planning based on CT scan of the knee is more inclined to the image-based option. Capece (27) et al. conducted a retrospective analysis of 300 patients who underwent knee arthroplasty using the Persona knee joint system. The conclusion showed that robotic technology allowed for a reduced level of constraint in the intraoperative choice between Posterior-Stabilized and Constrained Posterior-Stabilized liners compared with an imageless navigated procedure. Mancino (28) et al. and Rossi (29) et al. compared the accuracy of the planned implant positioning of a novel image-less robotic technique with an established navigated technique (NTKA), and the results showed that image-less had more advantages, especially in terms of the femur and tibia component alignment. So choosing which option is the best is a controversial issue that may be solved in the future by the development of artificial intelligence technology.

The integration of artificial intelligence (AI) in preoperative planning for total knee arthroplasty (TKA) has markedly improved both efficiency and accuracy. However, its potential is constrained by the current state of computer science technology (30). AI systems exhibit limited adaptability when confronted with complex cases, such as those involving severe bone defects, deformities, or ligament imbalances, and are susceptible to errors in automatic segmentation and planning (31). Current AI planning predominantly emphasizes the static alignment of prosthesis models and positions, lacking comprehensive dynamic analysis and functional reconstruction. Furthermore, these systems often neglect the holistic dynamic alignment of the ankle-knee-hip-pelvis-spine axis and postoperative lower limb stability, which are critical for ensuring long-term joint function (32).

The limitations of this study include the following: (1) Only 48 patients were selected (24 in each group), and the small number of patients might affect the statistical power and external validity of the results. Multicenter and large-sample randomized studies are needed to obtain more accurate and reliable results. (2) The motion of the KNEE joint is a comprehensive process involving ligaments, bones, and muscles. In this study, only CT data were imported into the AI KNEE software, and the involvement of soft tissues in knee joint motion was neglected. (3) Although the AI KNEE has more advantages in predicting prosthesis size, only the Attune PS prosthesis from Johnson & Johnson was selected, and the prosthesis type was relatively small. In future AI planning research, we will import various types of prostheses as much as possible. (4) The radiation dose and economic cost were greater for patients who underwent preoperative CT than for those who underwent x-ray fluoroscopy.

## Data Availability

The original contributions presented in the study are included in the article/Supplementary Material, further inquiries can be directed to the corresponding author.
